# CTCs detection from intraoperative salvaged blood in RCC–IVC thrombus patients by negative enrichment and iFISH identification: a preliminary study

**DOI:** 10.1186/s12894-021-00803-w

**Published:** 2021-06-10

**Authors:** Xiaoqing Zhang, Xiangyang Guo, Yanan Zong, Chuanya Xu, Jilian Wang, Bin Zhang, Chang Liu, Yueqing Gong, Lixiang Xue, Lulin Ma, Shudong Zhang, Yi Li, Hong Zeng

**Affiliations:** 1 Department of Anesthesia, Peking University Third Hospital, Huayuan 49# Rd, Haidian Dist., Beijing, China; 2 Biological Sample Bank, Peking University Third Hospital, Beijing, China; 3 Department of Urology, Peking University Third Hospital, Beijing, China

**Keywords:** Renal cell carcinoma, Inferior vena cava thrombus, Intra-operative cell salvage, Leukocyte depletion filter, Aneuploidy

## Abstract

**Background:**

Intra-operative cell salvage (IOCS) and leukocyte-depleted filter (LDF) are widely used and effective in saving blood. However, the safety issue concerning reinfusion of IOCS–LDF processed blood to renal cell carcinoma (RCC) patients with inferior vena cava (IVC) thrombus were inconclusive for fear of increased risk of cancer metastases. This study intends to analyze the circulating tumor cell (CTC) eliminating effect of IOCS–LDF in 5 RCC–IVC thrombus patients.

**Methods:**

A novel strategy integrating negative enrichment by immunomagnetic beads and immunostaining-fluorescence in situ hybridization with probes identifying aneuploid of 8 and/or 7 were used to detect CTCs from salvages blood. Blood samples were collected from 4 stages in each patient.

**Results:**

Of the 5 RCC patients, the number of CTCs decreased (from 3, 4, 10, 7, 3, respectively, to all zero) after IOCS–LDF treatment. The triploid of chromosome 7 and/or chromosome 8 were most common karyotype for RCC patients with IVC thrombus. Tetraploid of chromosome 8 occurred in only one sample and no polypoid (number of chromosome > 4) were found.

**Conclusion:**

IOCS–LDF might be a promising way of reducing of allogeneic product transfusion based on current preliminary outcome. More convincing conclusions are to be drawn with enlarged sample size and long-term follow-up for patients prognosis.

## Background

Renal cell carcinoma (RCC) ranks the sixth most frequently diagnosed cancer in men and the 10th in women worldwide. It is the 13th most common cause of cancer death worldwide according to data provided by the World Health Organization, as more than 140,000 RCC-related deaths yearly. The incidence rates of RCC is still rising, and this is partially because the increase in the imaging examination. Although most detected lesions are small tumors, locally advanced disease continues to be diagnosed in a notable proportion of patients, with up to 17% of patients harboring distant metastases at the time of diagnosis [1].

It is observed in 4–10% RCC patients who have a unique propensity for vascular invasion (into the renal vein and inferior vena cava) in the advanced stage [2]. Current guidelines recommend surgical excision of non-metastatic RCC with inferior vena cava (IVC) thrombosis in patients with acceptable performance status [3]. Patients with RCC–IVC tumor thrombus had a significant blood loss during operation despite improvements in surgical technique. The median estimated blood loss was 1900 (IQR 800–3300) ml. For patients with thrombus level ≤ 2 and ≥ 3 (Mayo-level), the blood loss were 1500 (IQR 600–2875) ml and 3000 (IQR 1400–5350) ml, respectively [4]. Massive transfusion of allogeneic blood may cause bacterial infection, allergic reactions, hemolytic reactions, transfusion-related risks of acute lung injury and viral infections. An association between perioperative transfusion of allogeneic blood products and risk for recurrence has been shown in colorectal cancer in randomized trials due to transfusion related immune modulation [5, 6]. It is concerned that allogeneic transfusion may result in immunosuppression and possible adverse effects on urological cancer recurrence [7–9].

Intra-operative cell salvage (IOCS) is of great significance in saving blood and offers an efficacious, alternative technique for blood replacement. Leukocyte-depleted filter (LDF) is normally used after IOCS washing and before infusion to remove nucleated cells such as bacteria and tumor cells. There were AAGBI safety guidelines in 2009, NICE guidelines in 2005, 2008 and 2015, UK Cell Salvage Action Group consensus in 2017, National Blood Authority in Australia in 2014, uniformly stated that despite theoretical risks and benefits, there is no conclusive evidence that cell salvage can induce metastases or affect cancer prognosis. The theoretical risk of inducing metastatic spread (unproven) is offset by reduced allogeneic transfusion and immunomodulation, which is proven [10]. However, concerns regarding the increased risk of cancer recurrence or development of metastases resulting from re-infusion of malignant cells renders the reluctance of adopting IOCS–LDF. At present, there were few studies on the safety assessment of recovered blood in patients with RCC–IVC thrombus. This study intends to analyze the tumor cell eliminating effect of IOCS–LDF in patients with RCC–IVC thrombus with a novel strategy integrating negative enrichment by immunomagnetic beads and immunostaining fluorescence in situ hybridization (NE-iFISH) with probes identifying aneuploidy.

## Method

### Patients selection

We recruited patients with RCC–IVC thrombus underwent open/laparoscopy radical nephrectomy and IVC tumor thrombectomy from January to December, 2018 in Peking University Third Hospital. Thrombus level was defined according to Mayo classification [11]. Only RCC–IVC thrombus (Mayo-levels II–IV) were included in the study. Excluding criteria: patients with blood diseases, infectious diseases, and those who refused to participate in the study. The study was approved by the hospital ethics committee (No. M2017296). Patients and their families all signed informed consent. Patients’ demographic information were recorded (Table 1).

**Table 1:** Patients’ demographic information, follow-up and tumor pathological classification

Case	Mayo-level grading	Surgical duration (min)	Tumor size (cm)	WHO/ISUP grading	Pathological diagnosis	20-mon follow-up	Tumor recurrence or metastasis
1	II	553	7.1 * 5.2 * 4.6	G2	ccRCC	Alive	None
2^a^	III	350	–	–	ccRCC	Dead	Preoperative lung metastasis
3	II	161	12 * 10 * 6	G3	ccRCC	Alive	None
4	IV	320	5 * 3 * 2.5	G2	ccRCC	Alive	None
5	II	540	9 * 5 * 4	G3	ccRCC	Alive	None

ISUP, International Society of Urological Pathology. G2, Tumour cell nucleoli conspicuous and eosinophilic at × 400 magnification and visible but not prominent at × 100 magnification; G3, Tumor cell nucleoli conspicuous and eosinophilic at × 100 magnification

*ccRCC* clear cell Renal Cell Carcinoma

^a^The patient underwent radical right nephrectomy for renal cancer 3 years ago. Now he was diagnosed as inferior vena cava thrombus with lung metastasis. The patient died of emphysema 16 months after surgery

### Anesthesia and surgical methods

General anesthesia was performed. Patients’ vital signs and the depth of anesthesia were closely monitored. The surgery of open/laparoscopy radical nephrectomy and IVC tumor thrombectomy were dictated by the extent and level of the tumor thrombus. The surgical technique has been described in detail by Berczi [12]. Surgical time, intraoperative blood loss, the amount of autologous blood recovery, and the amount of allogeneic blood transfusion were recorded. Recovered blood was not re-infused as this was not allowed by the ethical committee for fear of tumor dissemination and subsequent risk of metastatic disease.

### IOCS–LDF

The suction tube and blood reservoir of the IOCS machine (Cell Saver Elite, Haemonetics Corporation, MA, U.S.) was rinsed and pre-filled with 50–100 ml anticoagulant saline (heparin saline, 500 IU/ml). The negative pressure of the suction device is set at 120–150 mmHg. The anticoagulant drip rate was adjusted to about 1 drop/second, and the flow rate was adjusted according to the amount and speed of the bleeding. All intraoperative shed blood was recovered from the skin incision to tumor removal. Recovered blood was anti-coagulated and washed with sterilized saline (1500–2000 ml for 250 ml RBC). After washing, the recruited blood was filtered through PALL® leukocyte reduction filter (SB, Haemonetics Corporation, MA, U.S.). Blood products treated with IOCS–LDF are for research use only and are not returned to patients.

### Sample preparation

**Fig. 1:**
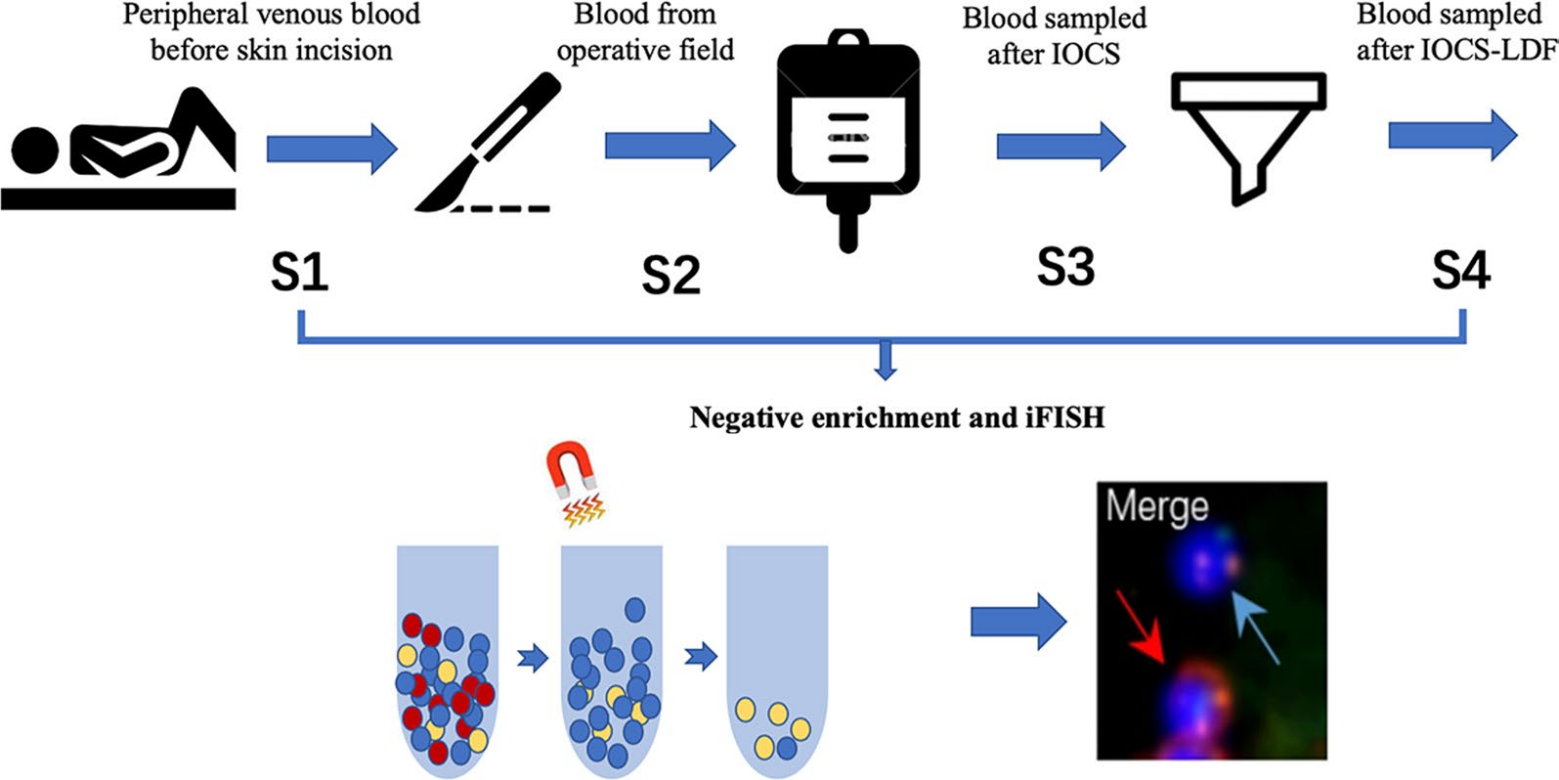
Schematic plot of sample collection and processing

Blood samples were collected intraoperatively at 4 different stages and sites (S1, peripheral venous blood from internal jugular vein before skin incision; S2, blood sampled in the vena cava around the tumor thrombus during surgery; S3, IOCS blood after washing and before LDF filtering; S4, blood sampled from post-LDF filtration). Each sample contains 4.0 ml blood (Fig. 1).

### Negative enrichment of CTC

The strategy of enrichment for circulating tumor cell (CTC) is performed according to manufacturer’s instructions. Briefly, 4.0 ml blood samples were lysed by RBC hypotonic hemolysis. Solution was centrifuged at 300 × *g* for 5 min. Then, the residue cell pellet was resuspended in PBS and subsequently incubated with anti-CD45 monoclonal antibody-coated magnetic beads for 30 min, followed by the separation of magnetic beads using a magnetic stand (Promega, Madison, WI, USA). Supernatants were subsequently subjected to identification.

### Immunocytochemistry staining of CTCs

The identification of enriched CTCs was performed by combing the chromosome enumeration using iFISH with chromosome 7 (CEP7, green) and 8 (CEP8, orange) centromere probes (Abbott Molecular Diagnostics, Des Plaines, IL, USA) and anti-CD45 monoclonal antibody (red) (CD45-FISH). Specimens were hybridized at 37 °C for 20 min in hybridizer (DAKO). Then they were washed in 50% formamide at 43 °C for 15 min, and subsequently immersed into 2*SSC and gradient alcohol again. Specimens were washed twice with 0.2% BSA and incubated with the CD45 mixture/2% BSA conjugated to Alexa Fluor 594 (Invitrogen) for 1 h. Afterward, samples were washed again with 0.2% BSA, and covered with DAPI which contained Vectashield mounting medium [13]. CTCs were examined and identified as hyperdiploid CEP8+/DAPI+/CD45−, hyperdiploid CEP7+/DAPI+/CD45− and hyperdiploid CEP7+, 8+/DAPI+/CD45− by two independent pathologists, while cells with CD45+ were differentiated as WBC.

### Statistical analysis

All data were recorded and compared among 5 RCC–IVC thrombus patients. Medians and quartiles were used to describe the amount of intraoperative blood loss, transfusion and recovered blood.

## Results

### Demographic information

**Fig. 2:**
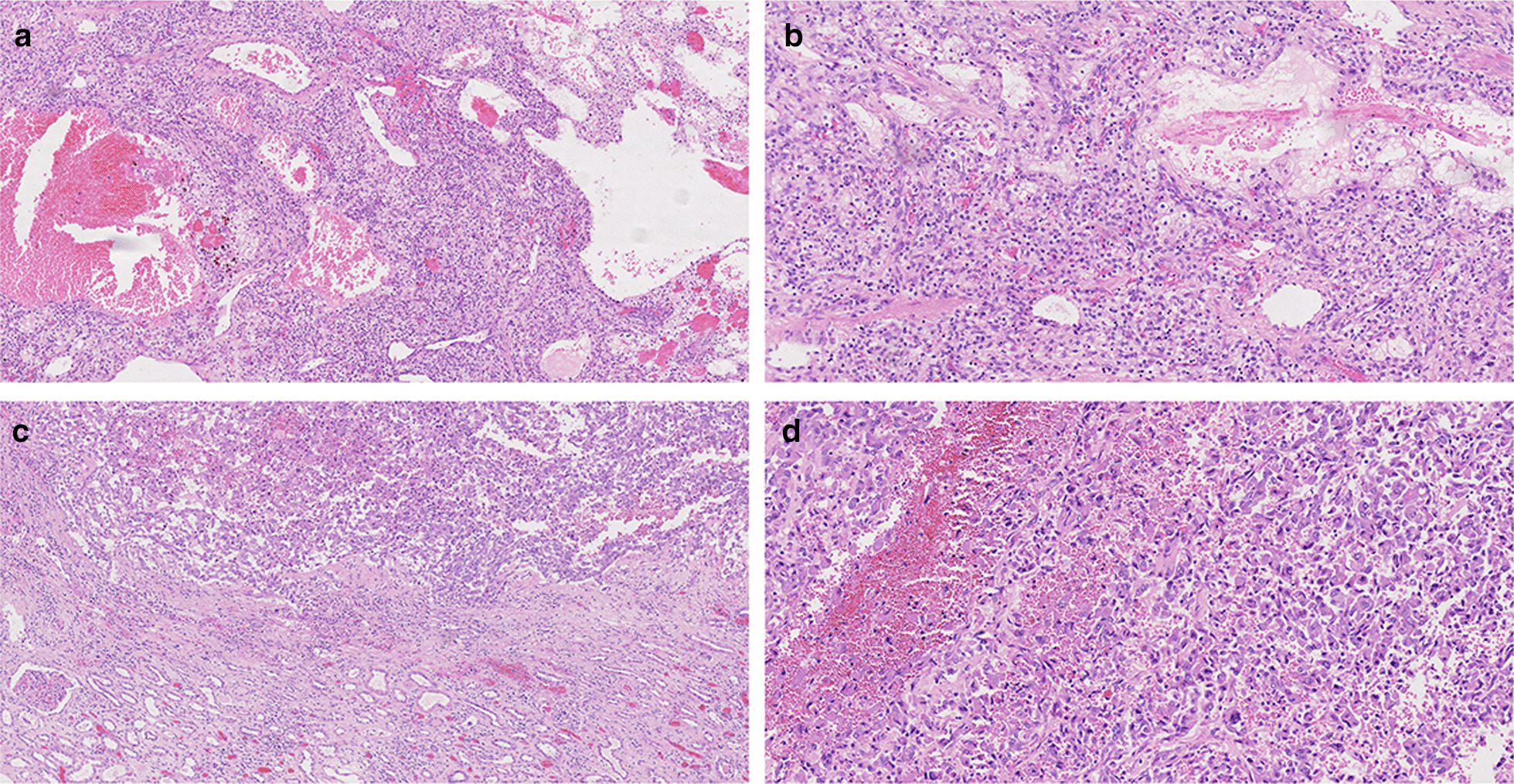
Pathological examination of the dissected renal tumor and thrombus. All five patients were diagnosed as ccRCC. **a**, **b** Histological examination showing the ccRCC as WHO/ISUP grade 2 (hematoxylin and eosin, original magnification: **a** × 100; **b** × 200). **c**, **d** Histological examination showing the ccRCC as WHO/ISUP grade 3 (hematoxylin and eosin, original magnification: **c** × 100; **d** × 200)

In the current study, a total of 5 patients with RCC–IVC thrombus were included for surgical treatment. Of all these 5 patients, one (case No. 2) underwent radical nephrectomy of the right kidney due to renal cancer in local hospital 3 years ago. The patient complaint shortness of breath recently and was admitted to our hospital. His lung CT revealed multiple nodules in both lungs, indicative of metastasis (4.1 cm * 3.8 cm). Abdominal CT revealed IVC thrombosis which was operated later and diagnosed from clear cell renal cell carcinoma (ccRCC) origin by pathological examination. The size of the renal tumors of the other four patients were 7.1 cm * 5.2 cm * 4.6 cm, 12 cm * 10 cm * 6 cm, 5 cm * 3 cm * 2.5 cm, 9 cm * 5 cm * 4 cm, respectively, and the WHO/ISUP grading system of the tumors were G2, G3, G2, G3, respectively [14] (Table 1). The pathological diagnosis of all 5 patients were ccRCC (Fig. 2). Post-operative follow-up showed the patient (case No. 2) with radical nephrectomy history died of emphysema 16 months after thrombectomy. Other 4 patients were closely followed up for at least 20 months and no cancer recurrence or metastasis were found till now.

### Intraoperative blood loss and recovery

A total of 5 patients (ASA II–III) were enrolled, including 1 female and 4 male patients, with an average age of 58.6 years. 3 patients (2 with Mayo-levels II, and 1 with Mayo-level IV) underwent open radical nephrectomy and IVC tumor thrombectomy, 1 (Mayo-level III) with open thrombectomy, and 1 patient (Mayo-level II) received laparoscopic radical nephrectomy and IVC tumor thrombectomy. Surgical duration was 335 (IQR, 320–540) min. The total intraoperative blood loss was 10,500 ml, and median amount of 2100 (IQR, 1500–2700) ml, with one patient (Mayo-level IV) lost 4000 ml blood. The total amount of transfused allogeneic red blood cells was 8800 ml, with median amount of 2000 (IQR, 1200–2000) ml. The total amount of transfused plasma was 2800 ml. One patient (Mayo-level II) who underwent laparoscopic radical nephrectomy and IVC tumor thrombectomy did not receive allogeneic blood transfusion due to small amount of blood loss. A total amount of 5150 ml of autologous blood, with median amount of 1000 ml (750, 1300) was recovered during surgery (Table 2). One patient (Mayo-level IV) experienced major bleeding and was managed with vasoactive drugs and massive blood product transfusion and fluid resuscitation. All patients’ vital signs were stable during hospitalization.

**Table 2:** Intraoperative blood loss and the transfusion

Case	Total blood loss (ml)	Infused RBC (ml)	Infused plasma (ml)	Recovered blood (ml)
1	2700	2000	0	1300
2	1500	1200	400	750
3	200	0	0	100
4	2100	2000	800	1000
5	4000	3600	1600	2000
Median	2100	2000	400	1000
IQR (ml)	1500–2700	1200–2000	0–800	750–1300

*IQR* inter quartile range

### Detection of tumor cells

20 blood samples from 5 RCC–IVC thrombus patients were studied. After excluding CD45+ leukocytes by immunomagnetic beads, the aneuploidy (abnormal chromosome numbers) of chromosome 8 and/or 7 were detected with iFISH examination. The aneuploidy of chromosome 8 or 7 include: trisomy 8, tetraploidy 8, polyploidy 8 (> 4), trisomy 7, tetraploidy 7, polyploidy 7 (> 4), aberrant number of both chromosome 8 and 7, and circulating tumor micro emboli (CTM). The negative expression of CD45 and the aneuploidy of chromosomes 8 and/or 7 (chromosome number > 2) were discriminated as CTCs (Table 3).

**Table 3:** CTC counts of aneuploidy of chromosome 8 and/or 7 from blood samples

Case	Sample	CTC counts									
		Triploid of chromosome 8	Tetraploid of chromosome 8	Polydiploid (> 4) of chromosome 8	Triploid of chromosome 7	Tetraploid of chromosome 7	Polydiploid (> 4) of chromosome 7	Polydiploidy chromosome of both 7 and 8	CTM	Sum	Total number
1	S1	3								3	
	S2										
	S3	1								1	
	S4										
											4
2	S1	1			2			1^a^		4	
	S2										
	S3				1					1	
	S4										
											5
3	S1	6	1		3					10	
	S2	1			1				1	3	
	S3										
	S4										
											13
4	S1	4			3					7	
	S2	3			1			1^a^		5	
	S3										
	S4										
											12
5	S1	1			1			1^a^		3	
	S2				1					1	
	S3										
	S4										
											5

Tumor cells were recognized and counted using immunostaining and fluorescence in situ hybridization technology. Only cells with CD45−/DAPI+/CEP8+ (> 2), CD45−/DAPI+/CEP8+ (> 2), and CD45−/DAPI+/CEP8+ and CEP7+ (hyperdiploidy) were identified as CTCs

Number 0 were not shown in the table

S1, peripheral venous blood sample from internal jugular vein before skin incision, indicative of the circulating tumor cells; S2, blood sampled in the vena cava around thrombus during surgery, indicating direct cells shedding in surgical manipulation; S3, blood after IOCS washing and before LDF filtering, which represents the tumor cell cleaning efficacy with only IOCS; S4, blood sampled after LDF filtration, meaning the actual final filtering result after IOCS–LDF

*CTM* circulating tumor microemboli

^a^Triploid of both chromosome 8 and 7

**Fig. 3:**
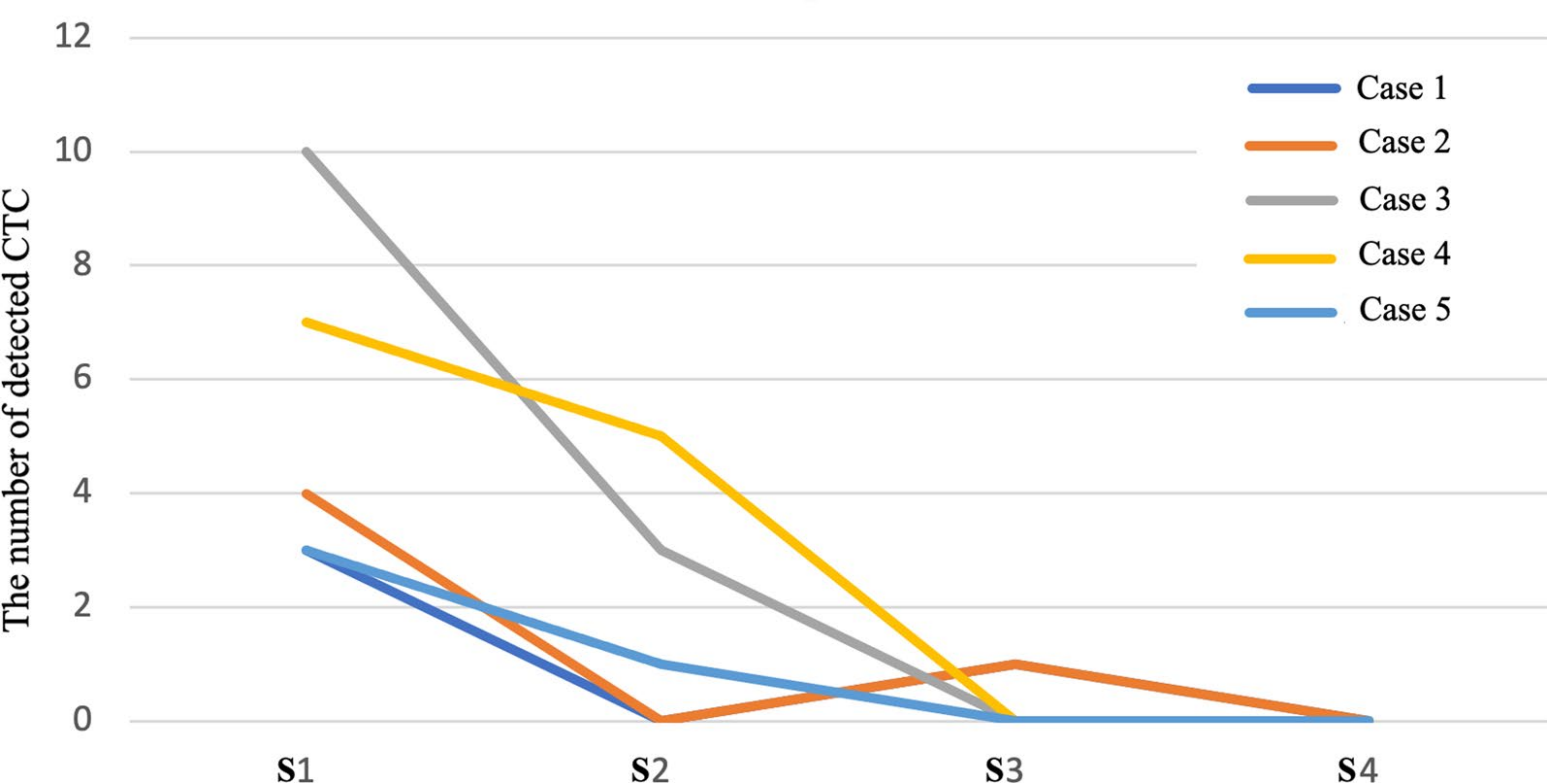
Detection and numeration of CTCs in 5 RCC patients at 4 different stages and sites. Significant decrease of CTC numbers were noted in all 5 patient. S1, peripheral venous blood sample from internal jugular vein before skin incision, indicative of the circulating tumor cells; S2, blood sampled in the vena cava around thrombus during surgery, indicating direct cells shedding in surgical manipulation; S3, blood after IOCS washing and before LDF filtering, which represents the tumor cell cleaning efficacy with only IOCS; S4, blood sampled after LDF filtration, meaning the actual final filtering result after IOCS–LDF

In all S1 samples, the number of tumor cells in the peripheral blood were 3, 4, 10, 7 and 3, respectively; and the number of tumor cells in the S2 sample were 0, 0, 2, 5 and 1, respectively; in S3 samples, the number of tumor cells decreased to 1, 0, 0, 0 and 0; and no tumor cell was found in any sample in S4 group (0, 0, 0, 0, 0) (Fig. 3).

In this preliminary study, most CTCs were of triploid of chromosome 8 (5/5 cases in S1, 2/5 cases in S2 and 1/5 cases in S3) or chromosome 7 (4/5 cases in S1, 3/5 cases in S2, and 1/5cases in S3), while only 1 case with tetraploidy of chromosome 8. The hyperdiploidy of both chromosome 7 and 8 were detected in 3 cases (2 cases of S1 and 1 case of S2). No tetraploidy of chromosome 7, or other aberrant chromosome number ≥ 4 were detected. (Table 4).

**Table 4:** Case number of aneuploidy of chromosome 7 and/or 8

Sample	Case numbers			
	Chromosome 8 triploidy	Chromosome 8 tetraploidy	Chromosome 7 triploidy	Chromosome 7 and 8 hyperdiploidy
S1	5	1	4	2
S2	2		3	1
S3	1		1	
S4				
Total	8	1	8	3

Triploidy of chromosome 8 were detected in 8 cases: 5/5 cases in S1, 2/5 cases in S2 and 1/5 cases in S3; Triploidy of chromosome 7 were detected in 8 cases: 4/5 cases in S1, 3/5 cases in S2, and 1/5cases in S3; The hyperdiploidy of both chormosome 7 and 8 were detected in 3 cases: 2 cases of S1 and 1 case of S2; Only 1 case with tetraploidy of chromosome 8; No tetraploidy of chromosome 7, or other aberrant chromosome number ≥ 4 were detected

## Discussion

### Aneuploidy of detected viable tumor cells

Intraoperative tumor metastasis occurred when large amounts of cancer cells were shed from primary tumor focus during surgical manipulation into the blood stream, becoming circulating tumor cells (CTC) and may target distant organs and develop metastatic tumors [15]. Reinfusion of viable tumor cells from intraoperative recovered blood poses potential risks of tumor dissemination and metastasis. However, due to scarce number of residual tumor cells from vast majority of leukocytes, capturing viable tumor cells from shed blood after IOCS–LDF treatment is difficult [16–19].

Currently, the majority of the methodology of detecting CTCs are restricted to the tumor cell density, size, and charge as well as biomarkers using antigen expression profiles and specific tumor antigen–antibody interactions to distinguish and isolate CTCs from other cells. However, a large quantity of primary CTCs are of smaller cell size (≤ WBCs) which makes it difficult to separate from WBC, and the rare CTCs are inevitability lost during filtration based cell size selection strategy [20]. Moreover, it is reported that positive EpCAM expression rate ranges 37–42.3% of the various cancers with FDA-approved Cell Search system. The invasive tumor cells tend to lose their epithelial antigens by the epithelial to mesenchymal transition process, which results in loss of EpCAM on CTCs and made the capturing of CTCs more difficult [21]. On the other hand, capturing non-tumor derived epithelial cells originated from inflammation, trauma, surgery and benign epithelial hyperplasia may cause false positive results [22]. For RCC, the expression of EpCAM was absent and there is no widely acknowledged specific cell surface marker for RCC detection[23, 24]. Therefore, a promising alternative approach of EpCAM-independent enrichment strategy has been introduced [25].

In this preliminary study, detecting aberrant chromosome is attempted as a way of capturing tumor cells in RCC patients. Aneuploidy is the abnormal alternation (either gain or loss) of chromosomes in a cell. It is estimated that 90% of solid tumors exhibit aneuploidy [20]. By integrating cellular and molecular approach of NE-iFISH, which is independent of cell size variation and free of anti-EpCAM perturbing. We could simultaneously co-detecting biomarker expression qualitatively and quantitatively, as well as discovering chromosome aneuploidy [13, 26].

**Fig. 4:**
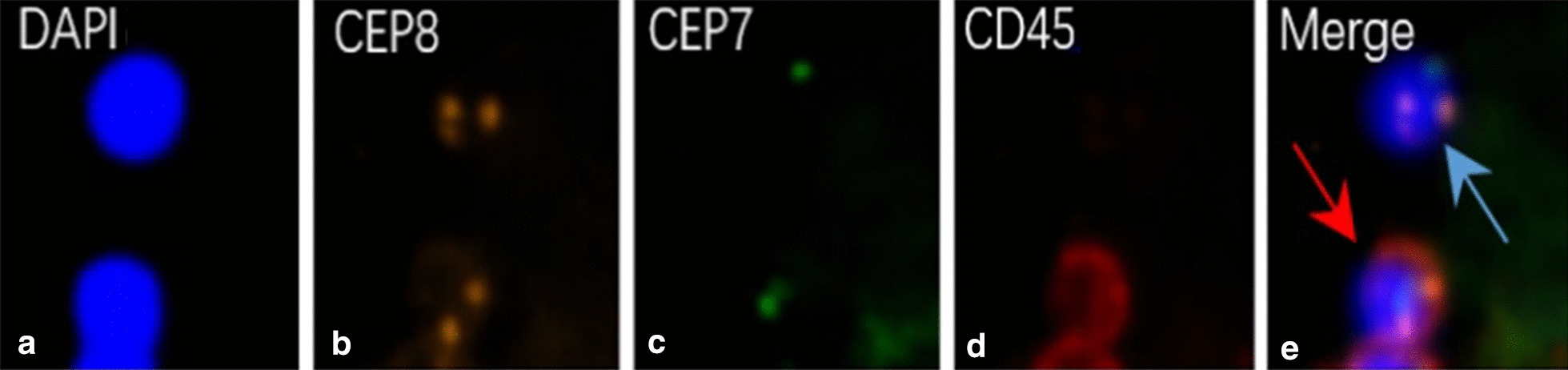
Detection and identification of RCC tumor cells by NE-iFISH. CTCs (blue arrow) were examined and identified as hyperdiploid CEP8+/DAPI+/CD45−, hyperdiploid CEP7+/DAPI+/CD45− and hyperdiploid CEP7+, CEP8+/DAPI+/CD45− by two independent pathologists. Cells with CD45+ were differentiated as WBC (red arrow). CEP, centromere polyploidy

It is reported that chromosome 7 and 8 abnormalities were detected in RCC cases. The positive rate of chromosome 7 abnormality ranges in 9.5–36%, and CTC detection rate using CEP 8 was 86.20% with a cut-off value of 1 CTC [27–30]. In this preliminary study, aneuploidy of chromosome 8 and/or 7 were analyzed and tumor cells were differentiated (Fig. 4). In this way, we could separate CTCs from recovered blood in RCC with IVC thrombus patients. To our knowledge, this is the first time that NE-iFISH was used to capture CTCs in the recovered blood of RCC–IVE thrombus patients with encouraging result.

It is interesting to note that CTCs recovered from intraoperative blood in ccRCC-IVC thrombus patients have the propensity of developing trisomy of chromosome 7 or 8. This chromosome karyotype was not reported before and deserve further exploration. It may be useful for future chromosome karyotype analysis and also for further study of the clinical significance in treatment efficacy of tumor recurrence, metastasis detection and prognosis.

### IOCS–LDF and RCC metastasis

Leukocyte depletion filter (LDF) is a filtering device based on a membrane-like filter material to remove leukocytes from blood. Its mechanism for removing tumor cells is physical interception and charge adsorption based on cell size. Kumar and colleagues revisited the safety of salvaged blood from the point of view of metastatic potential, and presented basic and applied science evidence regarding the innocuous nature of tumor cells that have been subjected to the cell salvage process. They believe that the current prevalent apprehensions on the usage of salvaged blood are ill-founded [31].

Radical nephrectomy and IVC tumor thrombectomy is widely acknowledged as the curative method for patients with RCC–IVC thrombus [32]. When the tumor thrombus extends to the right atrium, intraoperative establishment of extracorporeal circulation is required. Patients with Mayo-level III–IV often experienced life-threatening intraoperative major bleeding. As blood resource is limited, scheduled surgeries are always delayed. A recent meta-analysis has shown that the infusion of allogeneic blood during radical prostatectomy, radical nephrectomy and cystectomy lead to worsened prognosis for patients [33], and this may be related to transfusion-related immunomodulation [34]. The application of IOCS–LDF is an effective alternative for massive blood transfusion. A study concerning the IVC tumor thrombus extending to right atrium has shown that the use of cardiopulmonary bypass and cell-saver technique in borderline situations like combined oncologic and cardiovascular surgery without increasing the risk of hematogenous tumor dissemination. Postoperative cytological investigation showed that the tumor cells were only found on the internal surface of the heart–lung machine arterial filters. No distant metastasis was found in all surviving patients [35]. There were limited clinical study concerning the safety of returning shed blood to RCC–IVC thrombus patients, and the results were inconclusive [15, 36]. Other single center studies or meta-analysis of urological tumors showed that the use of IOCS with or without LDF did not increase the post-operative complication occurrences [37–39], or medical cost [39].

In the current study, we demonstrated with NE-iFISH method, all the tumor cells in 5 patients were completely removed after LDF treatment, and intraoperative IOCS–LDF usage could clear all tumor cells in RCC–IVC thrombus patients with high efficiency.

### IOCS–LDF for RCC–IVC thrombus tumor cell removal

In this study, 5 RCC–IVC thrombus patients were enrolled and 20 blood samples taken at different sites and stages during surgery were studied. Samples of S1 is indicative of the circulating tumor cells; S2 indicated peri-thrombus tumor cells shedding into inferior vena cava during operation; S3 represents the tumor cell cleaning efficacy with IOCS; and S4 is the actual final filtering result after LDF. Tumor cell numbers of all S4 samples were zero after IOCS–LDF treatment. This demonstrated that IOCS–LDF is effective in removing tumor cells. To our surprise, the number of tumor cells in internal jugular vein in S1 is most abundant (3, 4, 10, 7 and 3, respectively), while the cell number in S2 decreased (0, 0, 2, 5 and 1).

This might be explained by the solid texture of the IVC thrombus and its smooth surface which could extend itself way up to the atrium without being flushed away by blood flow in the inferior vena cava. The chances of tumor cell embolus detachment were not common. This also indicate that intraoperative exploration of the tumor thrombus does not necessarily result in significant shedding of tumor cells which might lead to tumor dissemination. Another important reason is related with patient blood management strategy. For RCC–IVC patients, considering the possible massive bleeding in the process of renal tumor and tumor thrombus separation and resection, anaesthesiologists would prefer to pre-infuse crystalloids and colloids to expand the blood volume and achieve tolerable hemodilution to reduce the red blood cells loss. This often results in hemodilution, which may lead to a relative decrease in the number of CTCs. In some conditions, apart from hemodilution, allogeneic blood products might also be pre-infused to prevent subsequent massive bleeding in a short time interval and causing violent hemodynamic fluctuation. Therefore, the number of CTCs might be reduced as a result of dilutional effect. In the current study, the S1 was sampled from peripheral venous blood in internal jugular vein before skin incision (when hemodilution was not achieved); while S2 was sampled in the vena cava around thrombus during surgery (when large amounts of crystalloid, colloids or blood products have been pre-infused).

Our study also demonstrated that IOCS alone is not enough for tumor cell depletion as CTCs were still detected in 2/5 cases in S3. The IOCS–LDF is efficient in depleting tumor cells from recovered blood as the number of CTCs reduced to 0 in all S4 samples. Due to limited sample size, further research with enlarge sample size is needed to verify the clearance ability of IOCS–LDF in RCC–IVC thrombus cases. Considering cell size differences, the application of IOCS–LDF to other tumor types may not achieve the equivalent effect.

Moreover, we also found that there is no close correlation between the amount of blood loss and the number of CTCs from the current study, which was also stated by Ernil Hansen in their research [40]. The number of CTCs were more prominent in patients with more advanced tumor stage and higher WHO-ISUP grading as well as Mayo-level grading. In case 3 and 4, the Mayo-level grading were II and IV, and the WHO-ISUP grading were G3 and G2, respectively. In case 3, the number of CTCs were 10 (S1) and 3 (S2), and the size of the dissected tumor is 12 cm * 10 cm * 6 cm; while for case 4, the number of CTCs were 7 (S1) and 5 (S2), with the size of the dissected tumor 5 cm * 3 cm * 2.5 cm. However, it is arbitrary to reach any definitive conclusion based on the limited sample size. Further research is warranted to verify this finding.

## Conclusion

There were no definitive guidelines or experts consensus to support the use of LOCS–LDF in RCC patients. There also lack laboratory and clinical evidence of safe reinfusion of autologous blood. In this preliminary study, CTCs of all samples decreased to 0 after IOCS–LDF in all five RCC–IVC thrombus patients. Which is encouraging. More convincing conclusions are to be drawn with enlarged sample size and discovery of more specific and accurate bio-markers for tumor cells originated from RCC–IVC thrombus.

## Data Availability

All data generated or analyzed during this study are included in this published article.

## Abbreviations

IOCS: Intra-operative cell salvage
LDF: Leukocyte-depleted filter
RCC: Renal cell carcinoma
IVC: Inferior vena cava
CTC: Circulating tumor cell
NE-iFISH: Negative enrichment by immunomagnetic beads and immunostaining fluorescence in situ hybridization
